# Aged Garlic Extract and Its Bioactive Molecules S-Allyl-Cysteine and S1-Propenyl-Cysteine: A Review Focusing on Evidences Supporting Their Use for Mitigating the Effects of Cigarette Smoking

**DOI:** 10.3390/molecules30173496

**Published:** 2025-08-26

**Authors:** Roberto Gambari, Alessia Finotti

**Affiliations:** Department of Life Sciences and Biotechnology, Ferrara University, I-44121 Ferrara, Italy

**Keywords:** natural products, cigarette smoke, inflammation, aged garlic extract

## Abstract

One of the major social issues worldwide is tobacco dependency and cigarette smoking (CS) abuse. Given the significant impact of cigarette smoking on human health and diseases, extensive tobacco use and cigarette smoking abuse are certainly a form of drug addiction and should be considered a serious threat to human health. Notably, healthcare spending attributable to cigarette smoking is very high. In this regard, a significant number of biomolecules of natural origin have been described as capable of mitigating the adverse effects of cigarette smoking. In this review, (a) we discuss the impact that the habit of smoking tobacco has on human health and (b) we describe products of natural origin capable of mitigating the effects of cigarette smoke. The conclusion of this review article is that the available information strongly indicates a possible use of the anti-inflammatory aged garlic extract (AGE) and its bioactive components for mitigating the detrimental effects of cigarette smoke on human tissues. The key reasons for proposing this application are that AGE and its key components S-allyl-cysteine and S1-propenyl-cysteine are potent anti-inflammatory agents, bind to Toll-like Receptor-4, inhibit Nuclear Factor-κB, inhibit the expression of pro-inflammatory genes, revert apoptosis induced by cigarette smoke in several cellular model systems and are strong inhibitors of Reactive Oxygen Species (ROS) formation.

## 1. Introduction

One of the major social issues worldwide is tobacco dependency and cigarette smoking (CS) abuse [1]. According to the publication *WHO global report on trends in prevalence of tobacco use 2000–2030* (16 January 2024, ISBN: 978-92-4-008828-3) [1], although the total number of tobacco users has declined steadily over the period 2000–2022, this number is still expected to be very high (around 1.20 billion) by 2030 [1,2]. Given the significant impact of cigarette smoking on human health and diseases [3,4,5,6,7,8,9], extensive tobacco use and cigarette smoking abuse are certainly a form of drug addiction and should be considered a serious threat to human health [1]. Notably, healthcare spending attributable to cigarette smoking is very high [10,11]. In order to limit tobacco use, several actions have been considered to help eliminate smoking [12,13,14,15], such as bans of tobacco advertising [16] and the introduction of taxes as a share of cigarette prices [17,18]. Despite these initiatives, the habit of smoking tobacco on a consistent basis is still a very significant social problem. In this regard, a significant number of biomolecules of natural origin have been described as capable of mitigating the adverse effects of cigarette smoking "in vitro" on cells and tissues and "in vivo" on complex organisms [19,20,21]. In this review, (a) we discuss the impact that the habit of smoking tobacco has on health and costs for national health systems, (b) we describe products of natural origin capable of mitigating the adverse effects of cigarette smoking and (c) we focus on the possible use of aged garlic extract (AGE) and its bioactive components for mitigating the adverse effects of cigarette smoking.

## 2. Impact of Cigarette Smoke on Human Health

### 2.1. Smoking and Human Diseases

**Figure 1:**
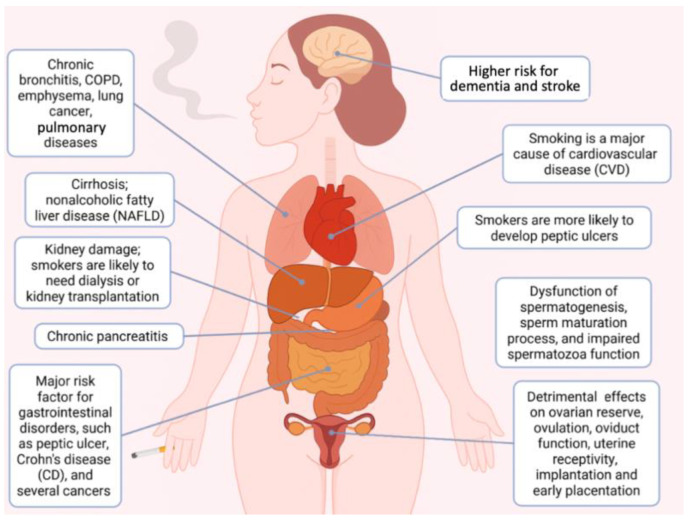
Human pathologies associated with cigarette smoking abuse. Picture created using Bio-Render.com (accessed on 16 July 2025).

Smoking causes cancer [22], heart disease [23], stroke [24], lung diseases [9,25], diabetes [26], chronic obstructive pulmonary disease (COPD) [27] and pancreatic diseases [28], as shown in Figure 1. Smoking is a particularly large problem in high-income countries, where cigarette smoking is the most important cause of preventable disease and death [29]. The impact of smoking is devastating on the individual level, considering that the life expectancy of those who smoke regularly is about 10 years lower than that of non-smokers [29]. The decline of cigarette smoking might be achieved through successful global health campaigns, including bans on tobacco advertising, the introduction of taxes on cigarettes and the development of plans to help people quit smoking. All these issues are discussed by Roser M (https://ourworldindata.org/smoking-big-problem-in-brief#) [accessed on 22 May 2025] [29]. For example, by taxing cigarettes very heavily, many governments made cigarettes much more expensive. Of course, reducing the affordability of cigarettes is one of the most important—and cost-effective—ways to reduce smoking and increase public health [29].

### 2.2. Smoking and Cancer

Smoking (and indirect smoking) causes or increases the risk for many types of cancer [30,31,32], including acute myeloid leukemia [33], bladder cancer [34], cervical cancer [35], colorectal cancer [36], esophageal cancer [37], prostate cancer [38], kidney cancer [39], laryngeal cancer and other throat cancers [40], liver cancer [41], lung cancer [42], oral cancer [43], pancreatic cancer [44] and stomach cancer [45].

In this respect, it should be underlined that tobacco smoking is associated in many cases with reduced efficacy or even failure of first-line cancer treatments; this causes incremental costs for the management of cancer patients [46]. In this respect, it is generally accepted that smoking seriously impacts health system costs, including those regarding cancer patients [47,48,49,50,51]. Accordingly, it is imperative that more stringent steps are taken to reduce the huge economic burden of human pathologies (including cancer) linked to smoking.

## 3. Mechanism(s) of Action of Cigarette Smoking: Inflammation 

The cellular and molecular mechanisms responsible for the solid interplay between cigarette smoke (CS) and inflammation have been reviewed by Lee et al. [52]. In this respect, it should be underlined that the identification of cellular, biochemical and molecular effects of CS is a key step for the identification of molecular targets for medical interventions. As a first consideration, we should mention that the several toxins and trace amounts of microbial cell components present in CS induce chronic inflammation [53,54,55]. In the CS-dependent activation of pro-inflammatory genes, several proteins play a crucial role and should be considered as possible biochemical targets for therapeutic intervention, among which is the Nuclear Factor-kB (NF-kB) pathway [56,57], associated with the activation of Toll-like Receptor-4 (TLR4) [58,59,60,61,62,63].

**Figure 2:**
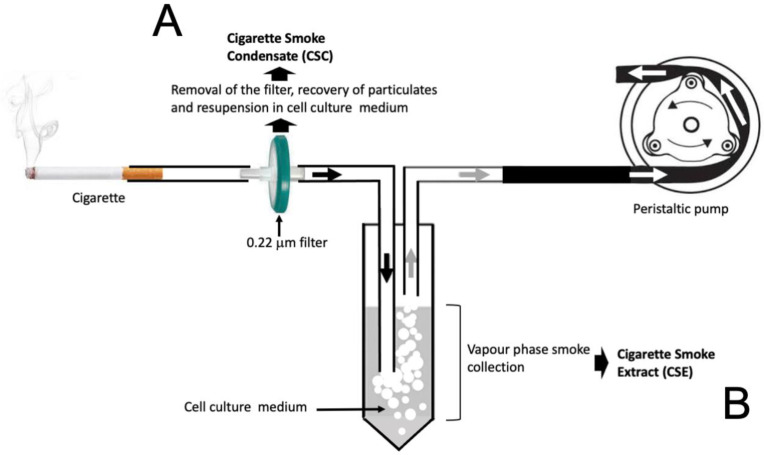
Scheme outlining the preparation of “Cigarette Smoke Concentrates” (CSCs) and “Cigarette Smoke Extracts” (CSEs), using information taken from Kim and Kim (2023) [70], Higashi et al. (2014) [71] and Wright (2015) [72]. In the representation here depicted, CSC is derived from the particulates trapped on the filter (**A**), whereas CSE is the resulting aqueous solution after bubbling the smoke through the medium (**B**).

Several experimental model systems are available to characterize the effects of cigarette smoke on cultured cell lines and the mitigation of these detrimental effects using natural products. Two are based on the production of “Cigarette Smoke Condensates” (CSCs) [64,65,66] and “Cigarette Smoke Extracts” (CSEs) [67,68,69]. Figure 2 reports a pictorial representation of the production of CSCs and CSEs starting from cigarette burning. 

The key step of CSC preparation is the trapping of the cigarette condensate in a 0.22 μm filter pad; then the cigarette smoke particulates are eluted using solvents, such as methanol, dimethyl sulfoxide (DMSO) or ethanol, recovered and transferred to tissue culture medium (CSC) for testing the CSC effects on cultured cells. Description of CSC preparation methods can be found in Kim and Kim [70] and in Mathewson [73]. CSE is an aqueous solution that contains toxic compounds produced by cigarette smoke. Therefore, CSE is useful to determine the effects of cigarette smoke on in vitro cultured cell lines. CSE can be prepared by collecting the smoke from a cigarette as shown in Figure 2. The cigarette smoke is “bubbled” in cell culture medium under a negative pressure generated by a peristaltic pump. The aqueous components are therefore diluted in the cell culture medium which, at the end of the procedure, is referred as “Cigarette Smoke Extract” (CSE). The parameters to be considered are the following: (a) number of the cigarettes; (b) volume of the cell culture medium and (c) flow rate generated by the peristaltic pump. Description of CSE preparation methods can be found in Amel Al-Hashimi et al. [69], Higashi et al. [71], Wight [72] and Agraval et al. [74]. A detailed protocol is available (https://dx.doi.org/10.17504/protocols.io.bnymmfu6; accessed on 22 May 2025).

In addition to using CSC and CSE, the effect of cigarette smoke in vitro can be assessed by direct exposure of cells or cellular tissues to cigarette smoke based on the air–liquid interface exposure [75]. In this respect, Singh et al. presented a perspective view of the challenges and opportunities of “Lung-on-Chip” technologies in studies focusing on cigarette smoking related in vitro inhalation toxicology [76]. With respect to the chemical composition of CSC and CSE, several studies are available [77,78,79,80,81]. In this respect, Kim et al. compared the volatile organic compounds (VOCs) of cigarette smoke condensate (CSC) and extract (CSE) samples [82]. The CSC sample mainly contained nicotine, nicotyrine and a lower relative amount of 1,2,3-propanetriol, triacetate, ethyl chloride and phenol [82]. The main composition of the CSE sample was different and contained acetonitrile, acetone, 2-hydroxy-2-methyl-propanenitrile and lower amounts of nicotine and nicotyrine [82]. Therefore, considering that the compounds in CSC and CSE are different, the effects (including toxicity) determined using CSC and CSE might differ. The following sections summarize the effects of CSC and CSE on biological functions, most of which are related to inflammation. 

### 3.1. Cigarette Smoking and Nuclear Factor-kB (NF-kB) 

Concerning the effects of cigarette smoking on the NF-kB pathway, Anto et al. found that the CSCmediated induction of cyclooxygenase-2 was associated with activation of NF-kB through phosphorylation and degradation of IkappaB(alpha) [56]. The proteasome-linked degradation of IkappaB(alpha) causes the translocation of NF-kB to the nucleus and the transcriptional activation of NF-kB-dependent genes [83,84,85,86,87]. Activation of NF-kB by cigarette smoke was also reported by Zhang et al. [88] and by Wang et al. [89]. Accordingly, products from the natural world targeting the NF-kB signaling pathway are of great interest and should be considered as potential anti-inflammatory agents for mitigating the effects of cigarette smoking [90,91,92,93]. For instance, Wang et al. reported that ghrelin inhibits interleukin-6 production induced by cigarette smoke extract (CSE) and this inhibition is based on targeting the NF-kB pathway [90]. In our own laboratory, we found that the NF-kB inhibitor corilagin attenuates the loss of cellular junctions induced by cigarette smoke in epithelial lung cells [93].

### 3.2. Cigarette Smoke and Toll-like Receptor-4 (TLR4) 

Nadigel et al. have reported that cigarette smoke increases TLR4 and TLR9 expression, thereby inducing increased cytokine production [61]. Interestingly, increased TLR4 expression was found in tissues of mice exposed to acute levels of cigarette smoke, and this was associated with lung inflammation [91,94]. Notably, elevated TLR4 and MMP-1 levels were found in lungs from smokers [94]. In conclusion, there is a general agreement on the fact that cigarette-smoking-related effects are mediated by activation of TLR-4 [58,59,60,61,62,63,94]. Accordingly, TLR4 inhibitors are expected to attenuate the acute cigarette-smoke-induced pulmonary inflammation [94,95]. As a representative and informative example, the TLR4 inhibitor TAK-242 (resatorvid) was administered by Wang et al. to mice exposed to cigarette smoke [91]. TAK-242 is a cyclohexane selected for inhibition of TLR4 [96]. It binds to the cysteine residue 747, preventing TLR4 binding with the toll-interleukin-1 receptor (TIR) domain-containing adaptor protein (TIRAP) [97] and downstream signal transduction. In the study by Wang et al., it was found to be very effective in mitigating the effects of exposure of mice to cigarette smoking. In fact, TAK-242 significantly decreased the accumulation of macrophages, neutrophils, lymphocytes and dendritic cells and the upregulation of IL-6, IL-8 and TNF-α in BAL fluid and lungs of the cigarette-smoke-exposed mice [91]. The results of this study demonstrated that the release of various inflammatory mediators is inhibited by TAK-242; notably, TAK-242 suppressed in lungs the expression of TLR4 and MyD88 as well as the activation of NF-κB [91]. These findings support the concept that TAK-242-mediated inhibition of cigarette smoke effects is associated with alterations of the TLR4/NF-κB signal pathway. Accordingly, TAK-242 can be proposed as a potent therapeutic agent in the treatment of cigarette-smoke-induced pulmonary inflammation. 

### 3.3. Cigarette Smoke and Increased Release of Pro-Inflammatory Proteins 

Fully in agreement with the effects of cigarette smoke on the TLR/NF-kB axis (see Section 3.1 and Section 3.2), cigarette smoke regulates the production of pro-inflammatory cytokines and chemokines by several in vitro cellular model systems [53,60,98,99,100,101,102,103]. Induced pro-inflammatory proteins include IL-6, TNF-α, IL-1β, IL-8, G-CSF, GM-CSF and MCP-1. For instance, Mio et al. reported that cigarette smoke induces IL-8 release from human bronchial epithelial cells [98]. Remarkably, cigarette smoke induced IL-8, but inhibits eotaxin and RANTES release from airway smooth muscle [104]. 

### 3.4. Cigarette Smoke and Apoptosis

Several reports are available on the induction of apoptosis with tobacco smoke and related products. Ramage et al. studied the induction of apoptosis using A549 lung epithelial cells as an in vitro model system [105]. In their study, A549 cells were treated with tobacco smoke condensate and apoptosis was measured morphologically following staining of cells with DAPI. In addition, activation of Bax-alpha, an early event in the apoptotic process, was measured; the results demonstrated that tobacco smoke was able to initiates apoptosis in A549 airway epithelial cells and this resulted in a cell detachment and full apoptosis. Cigarette-smoke-induced apoptosis was also demonstrated in alveolar epithelial cells [106], endothelial cells [107,108] and Raw264.7 cells [109]. Concerning cigarette-smoke-induced apoptosis, Banerjee et al. reported the very interesting observation that it was prevented by black tea in a guinea pig “in vivo” model system, associated with prevention of lung damage [110].

### 3.5. Cigarette-Smoke-Induced Formation of Reactive Oxygen Species (ROS) 

Cigarette smoke (CS) promotes ROS formation in different ways [111,112]. First of all, ROS, as well as radicals, are intrinsically present in CS [113,114,115]. In addition, CS constituents generate ROS through chemical reactions with biomolecules (quinones, redox-active metals, peroxy acids). For example, benzosemiquinones can penetrate the blood–air barrier and gain access to the blood circulation, thereby consistently producing superoxide through quinone redox cycling and forming adducts with biomolecules, such as hemoglobin and albumin [116,117]. Furthermore, CS stimulates cellular ROS sources (NOX, mitochondria, uncoupled eNOS) to enhance ROS production [112,118]. Finally, CS components (such as ethyl vinyl chetone, chrotonaldehyde, acrolein) disrupts the antioxidant system, aggravating ROS generation and functions [112,119,120].

## 4. Natural Products for the Mitigation of Toxic Biological Effects of Cigarette Smoke

The impact of natural products in preventing some of the more common detrimental effects of cigarette smoke is very high due to the low cost of these medical interventions, thereby allowing their use in developing low-income countries. A comprehensive review focusing on the protective effects of medicinal plants against cigarette smoke has been published by Tabeshpour et al. [19]. In this respect, Oriola and Oyedeji reviewed plant-derived natural products as useful agents against common respiratory diseases caused by cigarette smoke [121] (see Figure 1).

In this section, we will discuss some of the available examples showing the validated use of natural products for protecting cells or tissue against cigarette smoking and supporting the use of garlic-derived products (such as Aged Garlic Extract, S-allyl-cysteine and S1-propenyl-cysteine) for mitigating the effects of cigarette smoking both “in vitro” and “in vivo”. 

### 4.1. Silymarin 

Silymarin is a flavonolignan extracted from *Silybum marianum* (milk thistle seeds) reported to exhibit a broad spectrum of biological and pharmacological properties, including antioxidant, antiviral, anticancer and immunomodulatory activities [122]. Li et al. have reported that silymarin attenuates cigarette-smoke-extract-induced inflammation via simultaneous inhibition of autophagy and the ERK/p38 MAPK pathway in human bronchial epithelial cells “in vitro” [122]. In another study, the effects of silymarin were analyzed “in vivo”, demonstrating silymarin as a powerful inhibitor of airway inflammation induced by cigarette smoke in mice [123]. Silymarin pretreatment dampened the secretion of TNF-α, IL-1β and IL-8 in BALF. These results suggest that silymarin attenuated inflammation and oxidative stress induced by cigarette smoke. 

### 4.2. Eucalyptol

1,8-cineole (Eucalyptol), a naturally occurring compound derived from botanical sources such as *Eucalyptus globulus*, *Rosmarinus officinalis* and Camphor laurel (*Cinnamomum camphora*), has a long history of use in traditional medicine and exhibits an array of biological properties, including anti-inflammatory, antioxidant, antimicrobial, bronchodilatatory and analgesic effects [124]. Recent evidence has also indicated its potential role in managing conditions such as Alzheimer’s disease, neuropathic pain and cancer [125]. Eucalyptol suppresses lipopolysaccharide (LPS)-induced production of proinflammatory cytokines through an action on NF-κB, TNF-α, IL-1β and IL-6 as well as the extracellular signal-regulated kinase (ERK) pathway [125]. Eucalyptol was found to modulate CSE-induced human bronchial epithelial cell damage [126]. Accordingly, Yu et al. reported that treatment of rats exposed to cigarette smoke (CS) with eucalyptol mitigates CS-induced lung injury by suppressing ICAM-1 gene expression [127]. In addition, Kennedy-Feitosa et al. reported that eucalyptol inhibits lung inflammation and oxidative stress and promotes lung repair in mice following cigarette-smoke-induced emphysema [21,128]. 

### 4.3. Curcumin 

Curcumin is a constituent (up to ∼5%) of the traditional medicine known as turmeric [129,130]. Interest in the therapeutic use of turmeric and the relative ease of isolation of curcuminoids has led to their extensive investigation [130]. A comprehensive review on the protective effects of curcumin against cigarette-smoke-induced toxicity is available [131], and research articles reported that curcumin and liposomal curcumin inhibit cigarette-smoke-induced senescence and inflammation in human bronchial epithelial cells [132]. This effect is associated with a reduction in the expression of cigarette-smoke-extract-induced inflammatory markers IL-8 and IL-24 in vitro [133] through the modulation the PPARγ-NF-κB signaling pathway [134]. 

### 4.4. Taraxasterol

Taraxasterol is a pentacyclic-triterpene extracted from *Taraxacum officinalis* exhibiting anti-inflammatory properties [135]. Using lipopolysaccharide (LPS)-stimulated RAW264.7 cell as experimental model system, taraxasterol was reported as suppressing inflammatory cytokines, COX-2 and iNOS expression [136]. Xueshibojie et al. reported that taraxasterol inhibits CS-induced lung inflammation, ROS generation, IL-8 production, NF-κB activation, and TLR4 recruitment into lipid rafts [137].

### 4.5. Sulforaphane

The isothiocyanate sulforaphane (SFN) is one of the most abundant bioactive components of Brassicaceae (for example, broccoli) [138]. As extensively reported in previous studies, SFN exhibits a wide range of biological effects including anticancer, antioxidant, antimicrobial, neuroprotective, cardioprotective and anti-inflammatory activities [139]. As demonstrated by several studies, the anti-inflammatory activity of SFN is mediated by NF-κB inhibition [140,141]. Published research results are available demonstrating that sulforaphane protects alveolar epithelial cells against injury caused by cigarette smoke extract (CSE). In a first report, SFN was demonstrated to inhibit de novo synthesis of IL-8 and MCP-1 induced in human epithelial cells by CSE [142]. In another study, SFN was found to exhibit a protective role on CSE-exposed alveolar epithelial cells through an increase in Nrf2 expression [143,144].

### 4.6. Corilagin 

The polyphenol corilagin is extracted from different plants, including *Phyllanthus urinaria* [145], *Dimocarpus longan* [146] and *Geranium thunbergii* [147]. The beneficial effects of this natural compound in cardiovascular disorders, hypertension, thrombosis and atherosclerosis have been reported [145]. Zhao et al. have demonstrated that the anti-inflammatory properties of corilagin are based on a block of NF-κB activation and its nuclear translocation [148]. In agreement, corilagin decreases the production of pro-inflammatory proteins, such as TNF-α, IL-1β, IL-6, IL-8, iNOS and COX-2 [148]. In addition, corilagin inhibits ROS production from leukocytes as well as the formation of free radicals and lipid peroxidation in mitochondria [149,150]. In the study by Muresan et al., corilagin was found to mitigate the loss of cellular junctions induced in epithelial lung cells by cigarette smoke [93]. The results of this study demonstrated that CS induced the loss of cellular junctions in lung epithelium, possibly as a consequence of Cx-4HNE adduct formation, and corilagin was shown to be able to abolish these CS-induced alterations [93].

### 4.7. Trans-4,4′-dihydroxystilbene 

Trans-4,4′-dihydroxystilbene (DHS) is an analogue of the naturally occurring hydroxystilbene, resveratrol (3,4′,5-trihydroxystilbene, Resv), present in grape skins, red wines and grape juices. These molecules are widely accepted as very interesting because of their diverse pharmacological attributes [151]. Wang et al. found that 4,4′-dihydroxystilbene ameliorates cigarette-smoke-induced progression of chronic obstructive pulmonary disease via inhibiting oxidative stress and inflammatory response [152]. This study demonstrated that DHS attenuates the CS-induced pulmonary impairments through inhibition of oxidative stress and inflammatory responses targeting Nrf2 and NF-κB “in vitro” and “in vivo”, and could be developed into a preventive agent against pulmonary impairments induced by CS [152].

### 4.8. Other Example of Natural Products Against CS Effects

Several studies support the concept that natural products from medicinal plants alleviate cigarette-smoke-induced acute lung injury. Here are some examples. Liaqat et al. demonstrated that *Lavandula stoechas* significantly alleviates cigarette-smoke-induced acute lung injury via modulation of oxidative stress and the NF-κB pathway [153]. Similarly, Hussain et al. found that *Cichorium intybus L.* significantly alleviates cigarette-smoke-induced effects by lowering NF-κB pathway activation and inflammatory mediators [154]. Inhibition of the NF-kB pathway was also demonstrated as the mechanism of action explaining the anti-inflammatory and anti-oxidant properties of *Ipomoea nil (Linn.)* Roth [155]. Furthermore, examples of reversion of the detrimental effects of cigarette smoke were found using propolis [156], mate tea [157] and grape skin extracts [158]. 

## 5. Aged Garlic Extract and Its Bioactive Components: Candidates for Mitigating the Cigarette Smoking Effects

Among a large variety of natural products of biomedical relevance, garlic-based products have recently gained great attention [159,160]. Among these products, AGE (aged garlic extract) is well known and has been studied in detail [161]. AGE is a commercially available odorless preparation obtained by immersing fresh garlic in 15% aqueous ethanol solution over a prolonged period of time (up to 20 months) at room temperature [161,162,163,164,165]. This natural product has been shown to possess immunomodulatory and anticancer properties [160,161]. 

The chemical composition of garlic and AGE has been described by Kodera et al. [166], Borek [167], Ryu et al. [168] and El-Saadony et al. [169]. In particular, Kodera et al. focused on the number of the compounds present in AGE, their changes in content during the aging process, and their production mechanisms involving various chemical and enzymatic reactions 166]. The beneficial effects of garlic have been attributed to several bioactive compounds, including lipid-soluble allyl sulfur compounds (e.g., diallyl sulfide, diallyl disulfide and diallyl trisulfide) and water-soluble compounds such as S-allyl-cysteine (SAC), S-allylmercaptocysteine (SAMC) and S1-propenyl-cysteine (S1PC) [162,163,164,165,166]. In particular, water-soluble compounds (such as SAC and S1PC) are of interest, considering their high oral bioavailability, favorable pharmacokinetics and tissue distribution, which facilitate their clinical applications [170]. In this review, among the variety of chemical components [166], we focused on SAC and S1PC. These bioactive compounds might be extracted from AGE by unique manufacturing processes [165]. 

**Figure 3:**
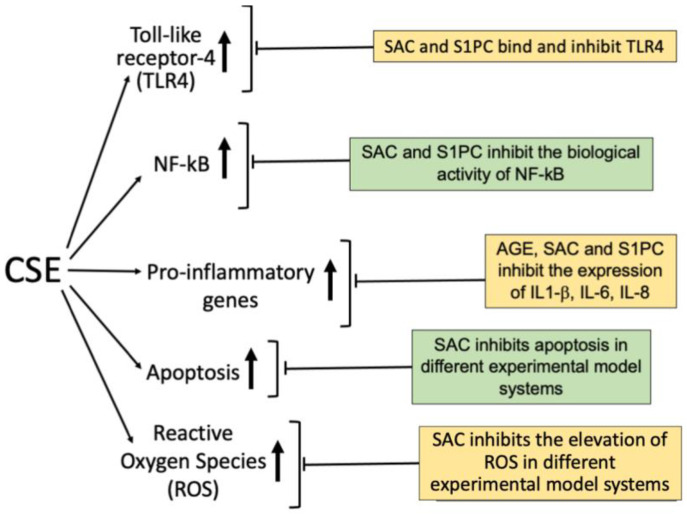
Biological features of AGE and AGE constituents SAC and S1PC supporting their use for mitigating the effects of cigarette smoke.

The anti-inflammatory Aged Garlic Extract (AGE) and its major bioactive components might be of great interest for mitigating the effects of cigarette smoking. The key reasons for proposing this application are summarized in Figure 3.

Notably, CS has been shown to induce a chronic inflammation. In this respect, several studies have revealed that AGE and its key components are potent anti-inflammatory agents, both “in vitro” and “in vivo” [171]. Furthermore, CS induced the TLR4/NF-kB pathway (see Section 3.1 and Section 3.2). In this respect, the AGE component S-allyl-cysteine (SAC) and S1-propenyl-cysteine bind to TLR4 [172,173,174] and inhibit NF-kB [171,175]. These findings should be further confirmed, since the cited studies have been performed using different methodological approaches and different cellular model systems, such as bronchial epithelial cells [171,172,173], chondrocytes [174] and T lymphoid cells [175]. 

A further consideration concerns the effects of CS on the expression of pro-inflammatory genes. CS induces IL-6, IL-8, IL-1β and several pro-inflammatory genes [60,98,99,100,101,102,103,104], and this effect appears to be selective. For instance, Oltmanns et al. reported that cigarette smoke induces IL-8, but inhibits eotaxin and RANTES release from airway smooth muscle [104].

We and several other research groups have clearly shown that AGE and the AGE components SAC and S1PC inhibit the expression of pro-inflammatory genes (such as IL-1β, IL-6, IL-8 and G-CSF) by targeting the TLR4 receptor [172,173,174] and the NF-kB pathway [171,175]. A consideration should also be made concerning the CS-mediated induction of apoptosis [105,106,107,108,109,110], as outlined in Section 3.4. Notably, Ramage et al. reported induction of apoptosis with tobacco smoke and related products in A549 lung epithelial cells in vitro [105]. In this respect, reports underlining the effects of garlic compounds on induced apoptosis in several cellular model systems are available [176,177,178,179]. Finally, CS induces Reactive Oxygen Species (ROS) [112,113,114,115], and this is strongly associated with oxidative stress and human diseases [180,181]. In this respect, S-allyl-cysteine is a strong inhibitor of ROS formation [182,183,184,185]. 

In this respect, we have to underline that few studies are available regarding the effects of garlic compounds on the biological effects caused by cigarette smoking. One of these studies has been reported by Hudlikar et al. in 2023 [64]. In this important study, the authors analyzed the effects of garlic compounds on transcriptomic changes induced in normal human lung epithelial Beas-2b cells by long-term exposure to cigarette smoke condensate (CSC). The effects of the organosulfur garlic compounds diallyl sulfide (DAS) and diallyl disulfide (DADS) were studied by Next Generation Sequencing (NGS) transcriptomic analysis. It was found that CSC regulated 1077 genes, including 36 genes modulated by DAS and 101 genes modulated by DADS [64]. The conclusion of this study was that CSC induces global gene expression changes which can be delayed with DS and DADS dietary phytochemicals [64]. This study therefore supports the concept that garlic compounds, including aged garlic extract, should be carefully analyzed for mitigation of the effects of cigarette smoke. 

The industrial interest in AGE and AGE-related products is documented by the fact that AGE is proposed and commercialized by several pharmaceutical companies, including for example Wakunaga Pharmaceuticals, Ltd (Hiroshima, Japan) (Kyolic^®^ Aged Garlic Extract), Evergreen Health Foods , Galway, Ireland (Quest Kyolic Aged Garlic Extract), Shaanxi Tianrun Phytochemical Co., Ltd, Xi’an, China (Garlic Extract, Allicin), Best Pharmacy.gr, Crete, Greece (Quest Kyolic Garlic) and Bizen Chemical Co., Ltd, Okayama, Japan (High SAC-Content Garlic). Notably, a trademark for S1-propenylcysteine (S1PC^TM^) has been recently obtained by Wakunaga Pharmaceuticals (registered on 9 July 2024; https://branddb.wipo.int/; accessed on 7 May 2025).

The industrial impact of AGE and AGE-related products is demonstrated by patents and patent applications focusing on these products. For instance, US8187654B2 (Title: Process for preparing aged garlic; Assignee: Blackgarlic Inc., Hayward, CA, USA) concerns a method of producing aged garlic in which its antioxidative capability is significantly increased as compared to that of raw garlic, which is used as a raw material. Methods for preparing aged garlic are described also in US20110293803, CN110623255A and EP1752051A1, as reported by Agostinelli et al. [171]. 

The possible transfer of the results concerning AGE and AGE-related products from bench to the bedside is supported by the growing number pf clinical trials. For instance, NCT1950646 (The Effect of AGE on the Immune System -EAGESIS II; sponsor University of Florida; last updated 26 February 2016) demonstrated that AGE consumption modulated immune cell distribution, prevented the increase in serum TNF-α and IL-6 concentrations and reduced blood LDL concentration in adults with obesity [186]. A further example is NCT03860350 (Aged Garlic Extract Study – AGE; sponsor Lund University Hospital; last updated 11 June 2019) demonstrating that AGE, supplemented with B vitamins, folic acid and L-arginine retards the progression of subclinical atherosclerosis [187]. Moreover, the same NCT03860350 trial found that AGE reduced IL-6 in females with a low risk of cardiovascular diseases [188]. Relevant to this review, NCT02019368 (A Randomized, Double-blind, Placebo Controlled, Crossover Study to Evaluate the Antioxidant Effect of Aged Garlic Extract in Heavy Smokers; sponsor Hiroshima University; last updated 19 August 2015) compared the oxidative status of heavy smokers with that of non-smokers and determined the antioxidant effect of aged garlic extract (https://clinicaltrials.gov; accessed on 18 July 2025). Inclusion criteria were smoking (at least 20 cigarettes per day) or non-smoking (more than 20 years). In this study, smoker subjects were programmed to take 1.5 g of the dietary supplement aged garlic extract in six capsules once a day for 4 weeks. The primary outcome measure was urinary 8-hydroxydeoxyguanosine (8-OHdG), taken every 4 weeks (overall 12 weeks). Despite the fact that information on the results obtained is still not available, the activation of this clinical trial (60 subjects were enrolled according to the 17 August 2015 status report) demonstrates the interest in studying AGE in clinical settings for heavy cigarette smokers. Based on the little information discussed in the present review, further pre-clinical studies and clinical trials are highly warranted. 

A final comment concerns the very interesting possibility that the best effects on CS-induced alterations occur when natural products are employed in combination. In the study performed by Reis et al., eucalyptol and curcumin used in in combination exhibited the highest efficiency in modulating cigarette-smoke-extract-induced human bronchial epithelial damage [126]. Therefore, combined use of eucalyptol and curcumin might be a potential therapeutic against smoking-induced lung diseases through antioxidant and inflammatory pathways [126]. Moreover, possible combinations using RNA/DNA-based drugs and natural products should be considered in the future. In this respect, aged garlic extract was recently proposed in combined treatments with microRNA miR-93-5p, previously demonstrated to inhibit TLR4, NF-kB and IL-8 gene expression [189]. This study provided preliminary evidence suggesting that the miR-93-5p-based miRNA therapeutics could be combined with the anti-inflammatory aged garlic extract (AGE) to more effectively inhibit IL-8 gene expression [189]. 

## 6. Conclusions

The conclusion of this review article is that the available information strongly indicates a possible use of the anti-inflammatory aged garlic extract (AGE) and its bioactive components S-allyl-cysteine (SAC) and S1-propenyl-cysteine (S1PC) for mitigating the detrimental effects of cigarette smoke on human tissues. Notably, the reported bioactive concentrations of AGE and AGE components are highly variable both “in vitro” and “in vivo”, depending on the biomarker analyzed, the methods employed for the analysis and the model system and administration protocol used. For instance, bioactive SAC concentrations were reported to vary between 10–200 μM [172,173,174,182] and 2–20 mM [164,165,179]. Therefore, preliminary studies are necessary to determine the optimal concentration to be used. In the representative clinical trials cited in this review, effective AGE concentrations employed varied between 250 mg daily [187] and 2400–3600 mg daily [186,188].

The key reasons for proposing AGE, S-allyl-cysteine (SAC) and S1-propenyl-cysteine (S1PC) for mitigating cigarette smoke effects are the following (summarized in Figure 3). First of all, AGE and its key components are potent anti-inflammatory agents, both “in vitro” and “in vivo”. Second, “in silico” and bio-molecular analyses indicate that the AGE bioactive components SAC and S1PC bind to TLR4, inhibit NF-kB and induce a decrease in the expression of pro-inflammatory genes. Furthermore, AGE and AGE components revert apoptosis induced by cigarette smoke in several cellular model systems. Finally, S-allyl-cysteine is a strong inhibitor of ROS formation. All the biological pathways mentioned are strongly induced by cigarette smoke in several cellular model systems (Figure 3). Experimental projects to verify this very interesting possibility are highly warranted, considering the impact of tobacco smoke on the health system (see Figure 1) [3,4,5,6,7,8,9]. It should be considered that healthcare spending attributable to cigarette smoking is very high [10,11] and several actions have been considered to help eliminate smoking [12,13,14,15], such as bans of tobacco advertising [16] and introduction of taxes as a share of cigarette price [17,18]. These smoking cessation interventions are important [14,15,16,190], even if difficulty in quitting smoking might be encountered [15,16]. In this context, strategies in preventing or mitigating the effects of tobacco abuse (such as those based on natural products, including aged garlic extracts and AGE components) are of great interest, considering the world-wide distribution of tobacco abuse [1].

## Data Availability

No new data were created or analyzed in this study; additional information will be shared upon request to the corresponding authors.

## Abbreviations

The following abbreviations are used in this manuscript:

CS	Cigarette Smoke
CSC	Cigarette Smoke Condensate
CSE	Cigarette Smoke Extract
VOC	Volatile Organic Compound
NF-κB	Nuclear Factor-kappa-B
TLR4	Toll-like Receptor-4
Nrf2	Nuclear Factor Erythroid 2-related factor 2
IL	Interleukin
ROS	Reactive Oxygen Species
AGE	Aged Garlic Extract
SAC	S-allyl-cysteine
S1PC	S1-propenyl-cysteine
SFN	Sulforaphane
DHS	Trans-4,4′-dihydroxystilbene
COPD	Chronic Obstructive Pulmonary Disease
CF	Cystic Fibrosis
BAL	Bronchoalveolar lavage
WHO	World Health Organization
